# Prenatal and postnatal lipid-based nutrient supplementation and cognitive, social-emotional, and motor function in preschool-aged children in Ghana: a follow-up of a randomized controlled trial

**DOI:** 10.1093/ajcn/nqy303

**Published:** 2019-02-05

**Authors:** Maku E Ocansey, Seth Adu-Afarwuah, Sika M Kumordzie, Harriet Okronipa, Rebecca R Young, Solace M Tamakloe, Brietta M Oaks, Kathryn G Dewey, Elizabeth L Prado

**Affiliations:** 1 Program in International and Community Nutrition, Department of Nutrition, University of California, Davis, CA; 2 Department of Nutrition and Food Science, University of Ghana, Legon, Ghana; 3 Department of Nutrition and Food Sciences, University of Rhode Island, Kingston, RI

**Keywords:** cognitive development, preschool development, lipid-based nutrient supplementation, multiple micronutrients, prenatal supplementation, postnatal supplementation

## Abstract

**Background:**

Adequate nutrition is necessary for brain development during pregnancy and infancy. Few randomized controlled trials of supplementation during these periods have measured later developmental outcomes.

**Objective:**

Our objective was to investigate the effects of provision of prenatal and postnatal lipid-based nutrient supplements (LNS) on child development at preschool age.

**Methods:**

We conducted a follow-up study of 966 children aged 4–6 y in 2016, born to women who participated in the International Lipid-Based Nutrient Supplements-DYAD trial conducted in Ghana in 2009–2014, representing 79% of eligible children. Women ≤20 weeks of gestation were randomized to daily LNS or multiple micronutrient (MMN) capsules during pregnancy through 6 mo postpartum or iron and folic acid (IFA) capsules during pregnancy and calcium placebo capsules during 6 mo postpartum. Children in the LNS group received LNS from 6 to 18 mo. Primary outcomes of this follow-up study were (1) a cognitive factor score based on a test battery adapted from several standard tests, 2) fine motor score (9-hole pegboard test), and (3) social-emotional difficulties (Strengths and Difficulties Questionnaire; SDQ). Eight secondary outcomes were calculated in specific domains (e.g., language, SDQ prosocial). Analysis was by a complete case intention to treat in a 2-group comparison: LNS compared with non-LNS (MMN + IFA).

**Results:**

Children in the LNS group had significantly lower social-emotional difficulties z-scores than children in the non-LNS group (adjusted for child age *β* = −0.12, 95% CI: −0.25, 0.02, *P* = 0.087; fully adjusted *β* = −0.16, 95% CI: −0.29, −0.03, *P* = 0.013). The effect of LNS on social-emotional difficulties score was larger among children living in households with lower home environment scores (*P*-interaction = 0.081). No other outcomes differed between the 2 intervention groups.

**Conclusions:**

Provision of LNS during the first 1000 d of development improved behavioral function, particularly for children from low nurturing and stimulation households, but did not affect cognition at preschool age in this setting. Trial Registration: clinicaltrials.gov, Identifier NCT00970866.

## Introduction

The initial years of life are critical for the formation of brain structure and capacity (1, 2). Neurodevelopmental processes occur rapidly during gestation and the first 2 y of life. Adequate nutrition is important to support these processes and for the long-term development of cognitive, motor, and social-emotional skills. In animal models, gestational and early postnatal nutrient deficiencies result in impairments such as reduced and truncated dendritic aborization, and alterations in myelin composition and synapse structure (2). In humans, many studies have shown associations between indicators of undernutrition and micronutrient deficiency, such as stunted growth and anemia, and developmental and cognitive function in early infancy and childhood (2). Results from randomized controlled trials (RCTs) of the effects of supplementation with specific micronutrients and fatty acids during pregnancy and infancy on child development have, however, been mixed (2–5).

Home fortification interventions including the use of multiple micronutrient powders and small-quantity lipid-based nutrient supplements (LNSs), have been evaluated to assess their potential to ameliorate the negative effects of undernutrition (6) on child growth and development in low- and middle-income countries. Although 5 RCTs of supplementation with LNS during the postnatal period (7–11) and 3 RCTs providing LNS during both prenatal and postnatal periods (12–14) have reported effects on developmental outcomes, results in early childhood up to age 2 y have been mixed, and none has examined longer-term developmental effects of LNS. Recently, we reported the results of the International Lipid-Based Nutrient Supplements (iLiNS) DYAD trial in Ghana showing that provision of LNS to women from pregnancy to 6 mo postpartum and to their infants from 6 to 18 mo positively affected linear growth (15), but did not affect motor, cognitive, or social-emotional development at age 18 mo (13). However, global behavioral developmental assessments before age 2 y may not be sensitive enough to detect effects. For example, a group of children who experienced thiamine deficiency in infancy did not show neurological symptoms at the time of deficiency, but showed language impairment at age 5–7 y (16). Similarly, in an RCT in the United States, infants who received formula containing DHA and arachidonic acid showed higher vocabulary and IQ scores at age 5–6 y than infants who received formula without these fatty acids, even though they did not differ in vocabulary or Bayley Scales of Infant Development scores at age 18 mo (17). These examples show that later effects of supplementation on cognitive and behavioral development may be observed, even if such effects were not detected on global developmental measures at a younger age. Here, we report a follow-up study assessing the effects of the intervention on cognitive, motor, and social-emotional development at age 4–6 y, using a comprehensive battery of cognitive, motor, and social-emotional tests adapted in the local Ghanaian setting. To our knowledge, our study is the first long-term follow-up of an RCT of both prenatal and postnatal LNS supplementation. We are aware of only 2 other RCTs that have conducted long-term follow-up assessment following nutritional supplementation in both the prenatal and postnatal periods, 1 of which randomized only 4 villages to intervention and control groups (18) and 1 of which had a high rate of attrition, re-enrolling only 55% of the original sample in the follow-up study (19, 20).

## Methods

### Study design and participants

The study reported here was a follow-up study of children and mothers who participated in the iLiNS-DYAD-Ghana randomized trial (15).

#### Design of the original trial.

Between 2009 and 2014, the iLiNS-DYAD-Ghana trial was conducted in semiurban communities in the Yilo and Manya Krobo Districts of the Eastern Region in Ghana, located about 70 km north of the capital, Accra. The trial tested the efficacy of providing 2 types of multiple micronutrient supplements compared with iron and folic acid, for preventing malnutrition in pregnant and postpartum women and their infants, and evaluated their effects on maternal nutritional status, child growth, micronutrient status, and neurobehavioral development at age 18 mo. At the time of the original trial, iron and folic acid supplementation was the standard practice and WHO and Ghana Health Service recommendation for antenatal care in Ghana, and multiple micronutrient supplements were already being evaluated as a likely alternative standard of care in many countries. A detailed description of the design and methods of the original trial (clinicaltrials.gov; NCT00970866) has been published elsewhere (15). In brief, pregnant women attending antenatal clinics in 4 health facilities in the area at ≤20 weeks of gestation were recruited into the study if they were ≥18 y old and agreed to participate by signing or thumb-printing informed consent after screening. Exclusion criteria were HIV infection, asthma, epilepsy, tuberculosis, any malignancy, known milk or peanut allergies, intention to move from the study area during the study period, unwillingness to receive fieldworkers or take study supplement, participation in another trial, or gestational age >20 wk before completion of the enrollment process.

A total of 1320 pregnant women were randomly assigned to 1 of 3 intervention arms daily from enrollment to delivery: *1*) 60 mg of iron plus 400 μg of folic acid [iron and folic acid (IFA) group: *n* = 441]; *2*) multiple micronutrient capsule containing 18 vitamins and minerals [multiple micronutrients (MMN) group: *n* = 439]; and *3*) LNS with similar micronutrients as the MMN supplement, plus other minerals and macronutrients (LNS group: *n* = 440) (15). The nutrient and energy contents of the supplements provided in the main trial are shown in **Table 1**. After birth, the MMN and LNS groups continued to receive the same supplements until 6 mo postpartum, whereas the control IFA group received calcium placebo capsule (200 mg/d) during that period. Children in the LNS group received LNS designed for children from 6 to 18 mo of age, whereas children in the other 2 groups received no supplement.

**TABLE 1:** Nutrient and energy contents of the supplements used in the International Lipid-Based Nutrient Supplement Dyad-Ghana randomized controlled trial in Ghana^1^

Nutrient	Chemical form used in supplement formulation	IFA	MMN	Maternal LNS	Child LNS
Ration per day	—	1 tablet	1 tablet	20-g sachet	20-g sachet
Total energy, kcal	—	—	0	118	118
Protein, g	—	—	0	2.6	2.6
Fat, g	—	—	0	10	9.6
Linoleic acid, g	—	—	0	4.59	4.46
α-Linolenic acid, g	—	—	0	0.59	0.58
Vitamin A, μg RE	Retinyl acetate	—	800	800	400
Vitamin C, mg	l-Ascorbic acid	—	100	100	30
Vitamin B_1_, mg	Thiamin hydrochloride	—	2.8	2.8	0.3
Vitamin B_2_, mg	Riboflavin	—	2.8	2.8	0.4
Niacin, mg	Niacinamide	—	36	36	4
Folic acid, μg	Pteroyl monoglutamic acid	400	400	400	80
Pantothenic acid, mg	Calcium pantothenate	—	7	7	1.8
Vitamin B_6_, mg	Pyridoxine hydrochloride	—	3.8	3.8	0.3
Vitamin B_12_, μg	Cyanocobalamin (0.1%)	—	5.2	5.2	0.5
Vitamin D, mg	Cholecalciferol (D_3_)	—	10	10	5
Vitamin E, mg	dl-α-Tocopherol acetate	—	20	20	6
Vitamin K, μg	Phylloquinone 5%	—	45	45	30
Iron, mg	Encapsulated ferrous sulfate	60	20	20	6
Zinc, mg	Zinc sulfate	—	30	30	8
Cu, mg	Encapsulated copper sulfate	—	4	4	0.34
Calcium, mg	Tricalcium phosphate	—	0	280	280
Phosphorus, mg	Dipotassium phosphate	—	0	190	190
Potassium, mg	Potassium chloride	—	0	200	200
Magnesium, mg	Magnesium citrate	—	0	65	40
Selenium, μg	Sodium selenite 1.5%	—	130	130	20
Iodine, μg	Potassium iodate	—	250	250	90
Manganese, mg	Manganese sulfate	—	2.6	2.6	1.2

^1^IFA, iron and folic acid capsule; MMN, multiple micronutrient supplement capsule; LNS, lipid-based nutrient supplement for pregnant and lactating women. Information from table previously published (13).

#### Follow-up study.

**FIGURE 1:**
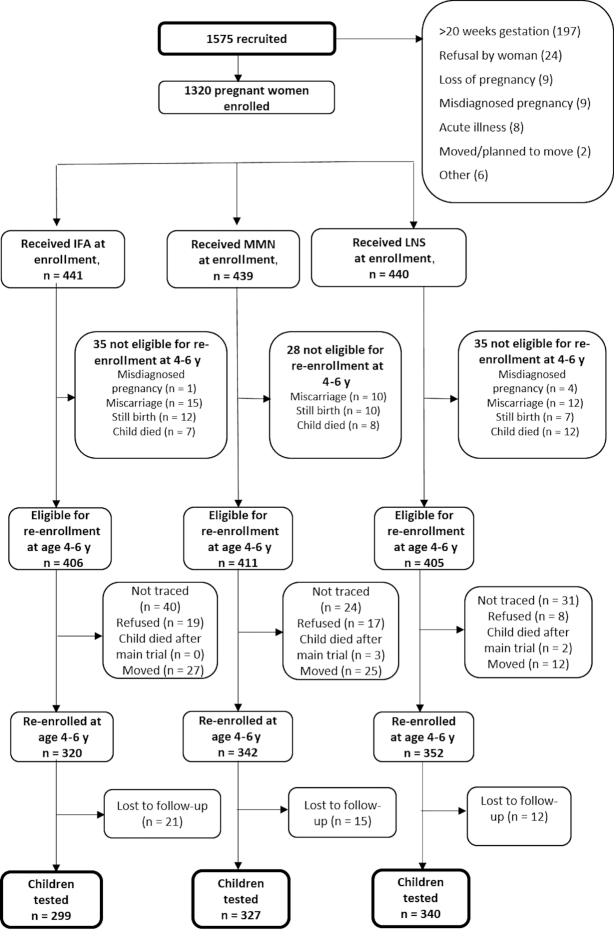
Study profile showing infants whose mothers were enrolled into the trial, and the reasons some infants were lost to follow-up. IFA, iron and folic acid; LNS, lipid-based nutrient supplement; LNS group, women received 20 g LNS daily during pregnancy and 6 mo postpartum; infants received 20 g LNS daily from 6 to 18 mo of age; MMN, multiple micronutrients; Non-LNS group, women received either IFA during pregnancy and placebo for 6 mo postpartum or MMN capsules during pregnancy and 6 mo postpartum. Infants did not receive any supplement. Groups shown are based on supplements women received at enrollment.

Between January and December 2016, all parents or caregivers of children who had participated in the iLiNS-DYAD-Ghana trial, including those who had relocated from the study site, for whom residential information and/or telephone numbers were available, were contacted for enrollment in the follow-up study. During this period, children who had been born during the trial were 4–6 y of age. **Figure 1** shows the trial profile. We re-enrolled 1014 children whose mothers or caregivers provided informed consent to participate, and obtained developmental data from 966 (79% of 1222 children whose vital status at 18 mo was alive or unknown). We excluded children if they had moved to a new location where a round trip to the study site would cost >60 Ghana cedis (∼US$15) at the time. In Ghana, these were children who could not make a round trip to the study site and complete the neurobehavioral assessments on the same day. Ethical approval for this follow-up study was obtained from the ethics committees of the University of California, Davis, the Ethics Committee for the College of Basic and Applied Sciences of the University of Ghana, and the Ghana Health Service Ethical Review Committee.

### Data-collection procedures

At enrollment into the original trial, maternal and household information including maternal age, birth order, education, and household assets was collected by trained fieldworkers using a questionnaire. Trained laboratory personnel measured maternal blood hemoglobin concentration at a scheduled clinic visit using a digital Hemocue (HemoCue model 301, AG, Switzerland) .

Field staff visited homes to explain the follow-up study, obtain consent for participation, and collect sociodemographic information. A second home visit was conducted typically within 7 d to obtain reports on child health and household investments and to invite participants to visit the test center for anthropometric, body composition, and neurobehavioral assessments. Phone interviews were conducted beforehand to ascertain that the child was in good health, and appointments were rescheduled in cases of reported illness from parents or caregivers. Procedures at the test center typically involved a 4-h visit including a snack break and lunch. Neurobehavioral assessment took about 1 h and 30 min.

For neurobehavioral assessment, mothers and children were assessed privately in a test room to reduce distractions. In addition to the snack break, children were given short breaks in between test sessions to reduce tiredness. Five trained data collectors who were blind to treatment group conducted the neurobehavioral assessments, with 1 data collector assessing 1–7 children/d. An additional home visit was conducted by 8 trained fieldworkers to interview parents or caregivers about the child's behavior and to assess nurturing and stimulation provided from the home environment. A laboratory visit by mothers and children was also done for biological sample collection. Data collection for a mother–child dyad in the follow-up study typically took 6–8 wk to complete.

### Developmental assessment measures

We assessed neurobehavioral development by several measures, as described in **Table 2**. Language ability was assessed by the body-part naming and identification and comprehension of instructions subtests of the Developmental Neuropsychological Assessment II (21). Preacademic skills were assessed using the Parent's Evaluation of Developmental Status developmental milestones test (22) administered by trained testers rather than by parent report. Executive function was assessed using the head–toe, delay of gratification, and visual search tasks. The head–toe task was drawn from the International Development and Early Learning Assessment (23), the delay of gratification from Noble (24), and the visual search task from the Supplementation with Multiple Micronutrients Intervention Trial (25). We assessed visuospatial ability using a block design test based on the British Ability Scales II pattern construction subtest (26) and the Wechsler Primary and Preschool Scale of Intelligence block design subtest (27). We assessed declarative memory using a paired associate memory task from Baddeley and colleagues (28). Motor function was assessed by the NIH Toolbox 9-hole pegboard test (29). We assessed social-emotional competence by caregiver interview using the Strengths and Difficulties Questionnaire (SDQ) (30, 31), which is a widely used brief screening questionnaire for child mental health problems in children aged 3–16 y. The SDQ comprises 25 items on psychological attributes divided into 5 subscales: emotional symptoms, conduct problems, hyperactivity/inattention, peer relations problems, and prosocial behavior. We calculated the total difficulties score, which assesses behavioral problems, as the sum of the first 4 subscales. We additionally assessed social-emotional development by direct observation using the Behavior Rating Scale adapted from the School Transition and Readiness study (32) based on the Preschool Self-Regulation Assessment data collector report (33). The tests were adapted to the local setting in Ghana and evaluated for test–retest and internal reliability through 2 rounds of pilot studies conducted in the study area before the follow-up study. For details of the test selection criteria and test adaptation process, see **Supplemental Methods**. The test–retest reliability of the adapted tests ranged from *r* = 0.61 to 0.94, and internal reliability (Cronbach's alpha) ranged from 0.61 to 0.9.

**TABLE 2:** Motor, cognitive, and social-emotional measures of assessment

Developmental domain	Developmental test	Test description and scoring
Motor		
Fine motor	NIH Toolbox 9-Hole Pegboard	We recorded the time required for children to accurately place and remove 9 plastic pegs from a pegboard, first with the dominant hand, followed by the other hand. The score was the mean time in seconds taken to complete the task with each hand.
Cognitive		
Language ability	Developmental Neuropsychological Assessment II Body Part Naming and Identification	Children were asked to say aloud or point to body parts on a line drawing of a person or on their own body. The 2 scores were the sum of body parts correctly named and identified.
	Developmental Neuropsychological Assessment II Comprehension of Instructions	Children were instructed to point to a picture, e.g., “Show me a puppy that is big and blue and happy.” The score was the number of items indicated correctly.
Visuospatial ability	Block Design	Children were asked to copy increasingly complex patterns of models built by the instructor, using wooden block in 30 s. The score was the number of structures correctly copied within the time limit.
Declarative memory	Paired-Associate Learning and Recall Task	Children were first taught new words for pictures of 8 objects and were asked to point to them as the instructor mentioned them aloud. They were later asked to recollect the words learned after a delay of median (IQR) 7 (6–11) min. We calculated the score as the mean number of correct responses on a set of 8 learning trials and 2 delayed recall trials.
Executive function		
Visual selective attention	Visual Search Test	Based on the Developmental Neuropsychological Assessment subtest adapted in the Supplementation with Multiple Micronutrients Intervention Trial, children were asked to identify all instances of a target picture (chicken or kitten) printed on a page with other distracter pictures as quickly as possible in 2 min. The score was the total time in seconds per correct target item identified.
Inhibition/reward	Delay of Gratification	The test was carried out 3 times for each child in between other tests. After each test, children were asked to make the choice of having 1 candy out of a tray of colorful candies immediately or having 2, 3, or 4 at the end of the second, third, and fourth test, respectively. Scoring was based on the number of candies chosen.
	International Development and Early Learning Assessment Head/Toes Test	Children were asked to inhibit the normal response to touch their head, when instructed to do so, by touching their toes instead. This was repeated 5 times, interchanging the touch head or toes instruction in a particular order. The score was the sum of correct responses.
Pre-academic skills	Parent's Evaluation of Developmental Status pre-academic subscale	In 14 items, children were asked to perform skills such as counting, reading aloud words, or identifying letters of the alphabet. The score was the total of correct responses.
Social-emotional		
Behavior regulation	Behavior Rating Scale	Based on the Preschool Self-Regulation Assessment Data Collector Report. The data collector assessed children's behavior or temperament at the test center and filled reports immediately after the test session for each child. Items on children's emotions, attention, behavior, defiance, noncompliance, and anxiety throughout the data collector–child interaction were scored on a Likert scale ranging from 0 to 3 (e.g., child is cooperative; child waits patiently for new tasks to begin) and some items were reverse-coded to minimize automatic responding. The score was the sum of data collector ratings on the 10-item scale.
Psychosocial and prosocial characteristics	Strengths and Difficulties Questionnaire	Parents or caregivers were asked during an interview to describe their child's behavior within the past 6 mo, based on a set of 25 questions divided between 5 scales: 1) emotional symptoms, 2) conduct problems, 3) hyperactivity/inattention, 4) peer relation problems, 5) prosocial behavior. Responses were scored on a Likert scale ranging from 0 to 2 (not true, somewhat true, and certainly true, respectively). Attributes 1–4 were summed up to generate a “Total difficulties score.”

### Additional measures at follow-up

We assessed the stimulation or caregiving available to children in the home environment using the Early Childhood version of the Home Observation for the Measurement of the Environment (HOME) Inventory (34), which we adapted to the local context. The test–retest reliability was 0.63.

Maternal depressive symptoms were assessed using the Edinburgh Postnatal Depression Scale, which has been validated among both postpartum and nonpostpartum women (35).

We used the General self-efficacy scale (36) to assess the general sense of perceived self-efficacy among mothers or caregivers.

### Training of data collectors and quality control

During the 1-y data-collection period, we conducted quarterly knowledge and practice-based evaluations to ensure that data collectors were standardized. At the beginning and during the last quarter of data collection, we evaluated the inter-rater agreement. For each of the 5 trained data collectors, we video-recorded 1 child testing session and 1 caregiver interview. All trained data collectors and their supervisors watched each video and independently scored the test or interview. For each data collector, the percentage of item scores that agreed with the supervisor was calculated. Overall agreement was calculated as the average agreement across data collectors for each test or interview. The inter-rater accuracy was high (>90%) for all tests except the visual search task (74%), due to slight differences between data collectors and their supervisor in regulating timers. The mean time to complete the task was 80 s, and the mean time difference between data collectors and the supervisor was 2.4 s.

### Sample size and statistical analyses

For the follow-up study, we hypothesized that: *a)* children in the LNS group will have better scores on motor, cognitive, and social-emotional function tests at preschool age than children in the MMN or IFA groups, and *b)* the percentage of children with severe and moderate to severe delays in motor, cognitive, and social-emotional development will be lower in the LNS group at preschool age than in the MMN or IFA groups. We estimated the effect size based on the number of mother–child pairs who completed the main trial (*n* = 1185) and presumed that attrition or refusal for the follow-up activities will be no more than 20% of that number. We expected that at least 948 participants would be involved in the follow-up at age 4–6 y, or approximately 316 per intervention group (LNS, MMN, IFA). With this sample size and a power of 80% at a 0.05 level of significance, we expected to be able to detect a difference of ≥0.25 SD in each of the 3 continuous primary outcomes.

We posted a statistical analysis plan with prespecified potential covariates and effect modifiers to the project website (www.ilins.org) before study investigators were unblinded to children's intervention group assignments. All analyses were conducted using SAS version 9.4 (SAS Institute).

We examined whether children in the 3 intervention groups were similar regarding a number of baseline and other characteristics, using ANOVA for continuous variables and chi-square for categorical (binary) variables. We also examined differences in baseline characteristics between children tested at follow-up and those lost to follow-up.

We calculated *z*-scores for each test score based on the distribution of scores in our sample. All *z*-scores were computed in 3-mo age bands, with a mean of 0 and a SD of 1 in each age band. Standard norming guidelines state that a sufficient sample size is 75–200 per age group. Age bands are expected to be smaller in the first year of age (1 mo) and larger at later ages (2–3 mo for toddlers and 6 mo or 1 y for school children) (37). Using 3-mo age bands, our sample included about 100 per age group, ranging from 4.25 y to 6.53 y.

The 3 primary outcomes were the cognitive, motor, and social-emotional domain scores. We calculated an overall cognitive factor score as the first factor of a factor analysis using the principal-axis factoring method including 7 outcome measures listed in Table 2: body part naming and identification, comprehension of instructions, preacademic skills, visual search, head–toe, block design, and paired associate memory scores, comprising all cognitive *z*-scores except the delay of gratification score, which was the only score that was not strongly associated with the other scores. The motor domain score was calculated as the mean of the NIH Toolbox 9-hole pegboard scores for the dominant and nondominant hands. The social-emotional domain score was the total difficulties score from the SDQ.

Eight secondary outcomes were also calculated. We calculated *z*-scores for the following: language, declarative memory, visuospatial ability, prosocial skills, delay of gratification, head–toe inhibition, preacademic skills, and behavior rating scale. We estimated the prevalence of severe and moderate to severe delay as the bottom 10% (lowest decile) and 25% (lowest quartile) of our sample, respectively, of scores in each domain. For the HOME inventory questionnaire, 3.6% of item scores were missing. The method described by Raghunathan and colleagues (38) was adopted to impute these missing items based on other items in the HOME.

We first tested the null hypothesis of no difference between the 3 treatment groups using ANCOVA for continuous outcomes (each domain *z*-score) and logistic regression for binary outcomes. For categorical multilevel or non-normally distributed count outcomes, we used ordinal probit regression and the negative binomial regression model, respectively, for analysis. For the ordinal probit regression, predicted probabilities were estimated to describe the relation between different levels of the response variable. Probabilities modeled were cumulated over the lower ordered values, assuming the same relation exists between sequential levels.

For all analyses, post-hoc pairwise comparisons of the 3 intervention groups were performed using Tukey–Kramer adjustment. We defined significant pairwise comparisons as *P* < 0.05. If there were no significant differences between the IFA and MMN groups, we combined these 2 groups into a non-LNS (control) group to estimate 2-group comparisons (LNS compared with non-LNS). The premise for combining the 2 control groups was that only the children in the LNS group were provided with supplements directly, from 6 to 18 mo of age.

We compared groups using 3 models. The first model was adjusted for child age at follow-up only (model 1). The second model was additionally adjusted for gender, developmental assessment data collector, and any of the following prespecified baseline variables that were significantly associated at the *P* < 0.1 level with the outcome in correlation analysis: maternal age, maternal education, maternal prepregnancy BMI, maternal hemoglobin concentration, household assets score, and parity (model 2). Third, we adjusted for any factors collected after enrollment (birth weight) or at follow-up (preschool quality, home stimulation score, maternal agency, and maternal depression) that were significantly associated at the *P* < 0.1 level with the outcome in correlation analysis (model 3). For any covariates that were collected after baseline, we first checked whether they were different between groups before including them in the model because they could be potential mediators.

We evaluated potential effect modification by 8 prespecified maternal (age, education, parity, hemoglobin concentration), household (household assets score, HOME score), and child (gender) variables for each outcome. We tested the interaction between each potential effect modifier and intervention group. Significant interactions (*P* < 0.1) were further examined with stratified analyses, or estimation of adjusted intervention group means at the 10th, 50th, and 90th percentile of the effect modifier, in order to understand the nature of the effect modification.

Maternal adherence to supplement use was determined by self-report, with data collected biweekly. In addition, fieldworkers collected any unused LNS sachets at each visit and reconciled the number of sachets remaining since the last visit. We calculated adherence as the percentage of follow-up days (e.g., during pregnancy or from enrollment to 6 mo postpartum) that the supplement was reportedly consumed. We conducted per protocol analyses in 2 ways: *1*) only including children of mothers who self-reported greater than or equal to 80% adherence (based on previous main trial analyses) to supplement consumption during pregnancy and *2*) only including children of mothers who self-reported greater than or equal to 80% adherence during the period of pregnancy up to 6 mo postpartum.

## Results

### Participants at follow-up

Out of 1320 women enrolled in the original trial, 1222 children were eligible for re-enrollment in this follow-up study, when excluding misdiagnosed pregnancies (*n* = 5), miscarriages and stillbirths (*n* = 66), and children who died before the end of the main trial (*n* = 27). We re-enrolled 1014 mother–child dyads at follow-up and obtained developmental data from 966 children (79% of 1222 eligible children and 73% of the 1320 women enrolled) (Figure 1). Children with neurobehavioral assessment data did not differ significantly in most background characteristics from those lost to follow-up. However, mothers of children included in this analysis were less likely to be nulliparous at enrollment (*P* = 0.032), and they had higher self-reported adherence to supplement use throughout pregnancy up to 6 mo postpartum (*P* < 0.0001) (**Supplemental Table S1**). The proportion of children lost to follow-up was significantly greater in the IFA group (32%) than in the LNS group (23%; *P* = 0.002) and significantly greater in the non-LNS group (IFA group + MMN group) (29%) than in the LNS group (23%; *P* = 0.018).

### Group characteristics comparisons

We did not find any significant differences between the IFA and MMN groups in any outcome, so we primarily report the 2-group comparisons combining the IFA and MMN groups, with analyses performed by a complete case intention-to-treat. The 3-group comparisons are presented in **Supplemental Table S2**.

The baseline and other selected maternal and child characteristics of the developmental sample are shown in **Table 3**. There were no significant differences between participants in the 2 intervention groups in 10 of the 12 background characteristics described in Table 3 (*P* > 0.05), but there were slight differences in 2 characteristics. Participants in the LNS group were from households with a lower mean asset score (*P* = 0.021) and were less likely to self-report adherence to supplement use during pregnancy through to 6 mo postpartum that was ≥80% than the non-LNS group (*P* = 0.003).

**TABLE 3:** Selected characteristics of women and children by intervention group at baseline and follow-up

	LNS^1^*n* = 340	Non-LNS^1^*n* = 626	
Variable	Mean ± SD (*n*) or % (*n*/total)	Mean ± SD (*n*) or % (*n*/total)	*P* value
Baseline maternal age, y	26.9 ± 5.5 (340)	26.8 ± 5.4 (626)	0.767
Pre-pregnancy BMI^2^, kg/m^2^	24.8 ± 4.4 (336)	24.4 ± 4.5 (613)	0.073
Gestational age at enrollment, wk	16.1 ± 3.3 (340)	16.1 ± 3.2 (626)	0.947
Baseline maternal education, y	7.6 ± 3.7 (340)	7.6 ± 3.4 (626)	0.466
Baseline maternal hemoglobin, g/L	111.2 ± 11.3 (340)	111.3 ± 12.4 (625)	0.898
Baseline household asset score^3^	−0.09 ± 1.0 (334)	0.06 ± 1.0 (619)	0.021
Nulliparous, %	32.4 (110/340)	32.0 (200/626)	0.943
Gestational age at delivery, wk	39.4 ± 0.10 (338)	39.3 ± 0.1 (623)	0.932
Child male, %	48.2 (164/340)	47.4 (297/626)	0.814
Child age at follow-up, y	5.0 ± 0.0 (340)	4.9 ± 0.0 (626)	0.096
Mean maternal adherence from pregnancy through 6 mo postpartum (percentage of supplements consumed)	67.2 (225/335)	76.3 (472/619)	0.003
Home stimulation score at follow-up	27.9 ± 4.5 (329)	27.9 ± 4.9 (599)	0.989

LNS; lipid-based nutrient supplement. Non-LNS; Iron & folic acid + multiple micronutrient capsules (control group).

^1^Results are based on ANOVA (SAS PROC GLIMMIX) or chi-square (SAS PROC FREQ).

^2^Estimated pre-pregnancy BMI was calculated from estimated pre-pregnancy weight (based on polynomial regression with gestational age, gestational age squared, and gestational age cubed as predictors) (39) and height at enrollment.

^3^Proxy indicator for household socioeconomic status constructed for each household based on ownership of a set of assets (radio, television, etc.), lighting source, drinking water supply, sanitation facilities, and flooring materials. Household ownership of this set of assets is combined into an index (with a mean of zero and SD of 1) using principal components analysis. Higher values represent higher socioeconomic status.

### Effects of the intervention

**Table 4** shows the mean motor, cognitive, and social-emotional development *z*-scores in the 2 intervention groups. In the age-adjusted analysis (model 1), there were no significant differences overall in any of the 3 primary outcomes measured at 4–6 y. However, the LNS group showed a trend for a lower mean social-emotional difficulties score than the non-LNS group (*P* = 0.087). With additional adjustment for baseline covariates (model 2: see footnote to Table 4), we found a significant difference between the 2 groups in social-emotional difficulties (*P* = 0.044). With adjustment for additional covariates collected after baseline (model 3: see footnote to Table 4), this difference remained significant (*P* = 0.013). The 3-group comparisons are presented in **Supplemental Table S3**. Results from a post-hoc exploratory analysis on the 4 subscales that make up the SDQ total difficulties score are reported in Supplemental Methods.

**TABLE 4:** Primary outcomes: motor, cognitive, and social-emotional *z*-scores at 4–6 y by intervention group^1^ and adjusted model

				Adjusted for child age at follow-up		Adjusted for baseline covariates		Adjusted for baseline and other covariates	
Developmental domain	*N* ^2^	LNS^3^Mean (95% CI)	Non-LNS^3^Mean (95% CI)	Difference in mean (95% CI)	*P* value	Difference in mean (95% CI)	*P* value	Difference in mean (95% CI)	*P* value
Cognitive *z*-score	951	0.03 (−0.06, 0.13)	−0.01 (−0.08, 0.06)	0.04 (−0.08, 0.16)	0.510	0.06 (−0.06, 0.18)	0.302^4^	0.05 (−0.08, 0.19)	0.432^5^
Motor *z*-score	963	0.00 (−0.10, 0.09)	0.00 (−0.11, 0.07)	0.00 (−0.13, 0.12)	0.935	0.06 (−0.12, 0.12)	0.978^6^	0.03 (−0.09, 0.16)	0.596^7^
Social-emotional difficulties *z*-score	959	−0.08 (−0.18, 0.03)	0.04 (−0.04, 0.12)	−0.12 (−0.25, 0.02)	0.087	−0.14 (−0.27, −0.00)	0.044^8^	−0.16 (−0.29, −0.03)	0.013^9^

LNS; lipid-based nutrient supplement. Non-LNS; Iron & folic acid + multiple micronutrient capsules (control group).

^1^We first tested the null hypothesis of no difference between the 3 treatment groups, and combined the iron and folic acid/multiple micronutrients groups because there were no significant differences between those 2 groups.

HOME, Home Observation for the Measurement of the Environment Inventory.

^2^Sample size based on model 1 adjusted for child age at follow-up.

^3^Results are based on ANCOVA (SAS PROC GLIMMIX).

^4^Adjusted for child age at follow-up, data collector, maternal education, maternal age, household asset score, and maternal hemoglobin.

^5^Additionally adjusted for exposure to multiple languages, type of preschool, and HOME score.

^6^Adjusted for child age at follow-up, data collector, and child sex.

^7^Additionally adjusted for exposure to multiple languages and HOME score.

^8^Adjusted for child age at follow-up, data collector, maternal education, prepregnancy BMI, maternal hemoglobin, maternal age, and household asset score.

^9^Additionally adjusted for maternal agency, maternal depression, and HOME score.

We found no significant differences between groups in any of the continuous secondary outcomes–-namely, language, visuospatial ability, declarative memory, preacademic skills, and behavior rating scale in unadjusted or adjusted analysis (**Table 5**). The incident rate ratio (95% CI) for the head–toe inhibition task was 1.03 (0.85–1.25), *P* = 0.738 (data not shown). For prosocial skills, the incident rate ratio (95% CI) was 0.99 (0.95–1.05), *P* = 0.968. This means that the mean counts of the LNS and non-LNS groups were almost equal for both tasks (data not shown). There were no significant differences across groups in response to the delay of gratification task (**Supplemental Table S4**).

**TABLE 5:** Secondary outcomes: Selected developmental outcomes at 4–6 y by intervention group^1^ and adjusted model

				Adjusted for child age at follow-up^2^		Adjusted for baseline covariates^3^		Adjusted for baseline and other covariates^4^		
Developmental domain	*N* ^5^	LNS^6^ mean (95% CI) or OR (*n*/total)	Non-LNS^6^ mean (95% CI) or OR (*n*/total)	Difference in mean or OR (95% CI)	*P* value	Difference in mean or OR (95% CI)	*P* value	Difference in mean or OR (95% CI)	*P* value	Covariates
Language *z*-score	963	0.00 (−0.08, 0.09)	0.00 (−0.06, 0.06)	0.00 (−0.10, 0.11)	0.936	0.02 (−0.08, 0.12)	0.702	0.01 (−0.09, 0.11)	0.796	A, B, F, G, H, M, N
Visuospatial *z*-score	963	−0.02 (−0.08, 0.04)	0.00 (−0.04, 0.04)	−0.02 (−0.09, 0.06)	0.675	−0.02 (−0.09, 0.05)	0.612	−0.01 (−0.09, 0.08)	0.853	B, C, G, I, K, N
Preacademic *z*-score	958	−0.01 (−0.12, 0.10)	0.00 (−0.08, 0.08)	−0.01 (−0.15, 0.12)	0.852	0.00 (−0.12, 0.13)	0.965	−0.01 (−0.15, 0.14)	0.927	A, B, C, D, E, G, H, I, J, K, M, N, O
Declarative memory *z*-score	958	0.04 (−0.06, 0.14)	−0.03 (−0.10, 0.04)	0.07 (−0.06, 0.19)	0.285	0.08 (−0.04, 0.20)	0.209	0.04 (−0.09, 0.17)	0.568	A, B, E, F, H, I, N
Behavior rating *z*-score	962	0.00 (−0.11, 0.10)	0.0 (−0.08, 0.08)	−0.01 (−0.14, 0.12)	0.910	−0.01 (−0.14, 0.12)	0.922	−0.01 (−0.15, 0.13)	0.884	B, G, H, I, M, N
Cognitive lowest decile	951	10.2 (34/335)	10.1 (62/616)	0.99 (0.64, 1.55)	0.972	0.95 (0.60, 1.51)	0.834	1.05 (0.59, 1.86)	0.880	B, E, G, J, L, N
Cognitive lowest quartile	951	24.5 (82/335)	25.3 (156/616)	0.95 (0.70, 1.30)	0.761	0.93 (0.67, 1.28)	0.637	1.01 (0.71, 1.44)	0.952	A, B, C, E, H, I, M, N
Social-emotional problems highest decile	958	8.9 (30/336)	10.9 (68/622)	0.80 (0.51, 1.26)	0.332	0.74 (0.47, 1.18)	0.208	0.64 (0.39, 1.06)	0.085	H, M, N, O
Social-emotional problems highest quartile	958	20.8 (70/336)	27.8 (173/622)	0.68 (0.49, 0.93)	0.016	0.65 (0.47, 0.90)	0.010	0.61 (0.43, 0.88)	0.008	A, B, C, E, H, M, N, O
Motor lowest decile	963	11.2 (38/338)	9.4 (59/625)	1.23 (0.80, 1.89)	0.355	1.24 (0.80, 1.89)	0.333	1.23 (0.75, 2.02)	0.404	G, I, N, O
Motor lowest quartile	963	25.4 (86/338)	24.8 (155/625)	1.02 (0.75, 1.39)	0.879	1.02 (0.75, 1.39)	0.885	1.03 (0.73, 1.46)	0.853	B, G, I, N, O

LNS; lipid-based nutrient supplement. Non-LNS; Iron & folic acid + multiple micronutrient capsules (control group).

^1^We first tested the null hypothesis of no difference between the 3 treatment groups and combined the iron and folic acid/multiple micronutrients groups because there were no significant differences between those 2 groups.

^2^All models were adjusted for child age at follow-up.

^3^Baseline covariates = A–F.

^4^Other covariates collected after baseline = G–O. A = maternal age; B = maternal education; C = maternal hemoglobin; D = maternal prepregnancy BMI; E = household asset score; F = primiparity; G = child sex; H = data collector; I = exposure to multiple languages by 18 mo; J = type of preschool; K = teacher's education; L = mean time in instructions at preschool; M = maternal depression; N = home stimulation score; O = maternal agency.

^5^Sample size based on model 1 adjusted for child age at follow-up.

^6^Results are based on ANCOVA (SAS PROC GLIMMIX).

Estimating the percentage of children in our sample with severe (lowest decile) or moderate-to-severe delay (lowest quartile), we found no significant differences between groups in any developmental outcome, except for a lower percentage of children in the LNS group than in the non-LNS group in the highest quartile of the social-emotional difficulties score (LNS 20.8%; non-LNS 27.8%; *P* = 0.016). This difference remained significant in model 2 and model 3 (Table 4). In the 3-group comparisons, these differences were also consistent across models (data not shown). In the per protocol analysis when including only children of mothers who self-reported ≥80% adherence to supplement consumption during pregnancy or during pregnancy through 6 mo postpartum, the pattern of results was consistent with that shown for the full sample, with significant effects on social-emotional difficulties but not on cognitive and motor scores (data not shown).

### Effect modification

**FIGURE 2:**
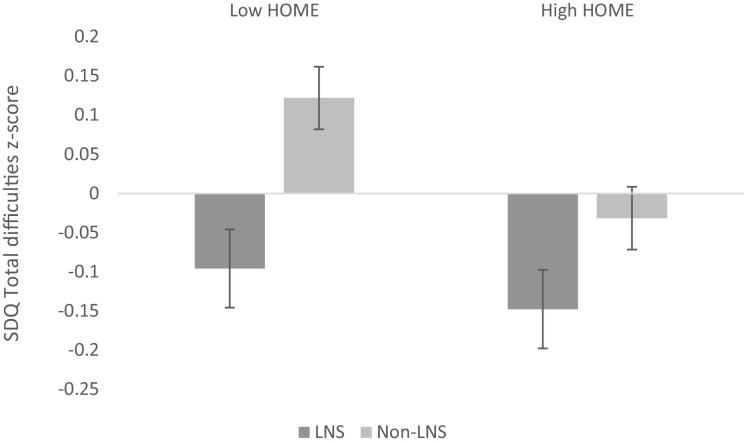
Mean social-emotional difficulties score by intervention group and HOME score. Error bars show the standard error of the mean. High HOME, HOME score above the sample median; HOME, Home Observation for the Measurement of the Environment Inventory; LNS, lipid-based nutrient supplement for mothers and children; Low HOME, HOME score below the sample median; Non-LNS, iron and folic acid or multiple micronutrient capsules for mothers only (control group). *P* for interaction between HOME score (categorized by the median population score as low HOME or high HOME score) and intervention group = 0.081.

For each of the 8 continuous outcomes measured, we examined 8 potential effect modifiers: child sex, HOME score, household asset score, and the following maternal factors collected at baseline: age, prepregnancy BMI, education, hemoglobin level, and parity. Six out of 64 (9%) interactions between the effect modifier and group were found to be significant at *P* < 0.1, which is the proportion that would be expected due to chance. For any effect modifier, the maximum number of interactions found to be significant was 3 out of 8 outcomes; thus, none of the variables was a consistent effect modifier across outcomes. The interaction between intervention group and HOME score was significant for the primary social-emotional development outcome that significantly differed between groups (*P* interaction = 0.081). The effect of the LNS intervention on the behavioral problem *z*-score was larger among children from households with HOME score below the median (*β* = 0.22 SD ± 0.09; *P* = 0.019) than for children from households with higher HOME scores (*β* = 0.12 SD ± 0.09; *P* = 0.204) (**Figure 2**).

## Discussion

In this randomized trial, children who received LNS from 6 to 18 mo of age, and whose mothers received LNS during pregnancy and through 6 mo postpartum, had significantly lower mean scores for behavioral problems, indicating better social-emotional behavior at 4–6 y of age than control children whose mothers received IFA or MMN. Intervention group differences in behavioral problem scores were larger among children who received less nurturing and stimulation from the home environment. Cognitive and motor scores at age 4–6 y, the other primary outcomes, did not differ significantly between intervention groups, nor did any secondary outcomes, which included language, visuospatial ability, declarative memory, inhibitory control, preacademic skills, behavior rating scale, prosocial skills, and delay of gratification scores.

The strengths of this study include the randomized study design, which allowed us to examine long-term effects of nutrient supplementation during most of the first 1000 d from conception to age 24 mo. We used a wide range of carefully selected tests adapted to the Ghanaian context, which showed high reliability in the local context. We implemented standardized test procedures and rigorously trained a team of data collectors who achieved high inter-rater agreement (>90%). Developmental outcomes were collected from 79% of children eligible for the follow-up from the main trial, indicating a relatively low attrition rate. One limitation is that, during the main trial, participants could not be blinded to receipt of LNS compared with MMN or IFA because of the differences in their appearance, although data collectors who conducted the neurobehavioral assessments at follow-up and data analysts were kept blinded. Lack of blinding of parents or caregivers could have biased their responses for the neurobehavioral assessments based on the caregiver report, i.e., the SDQ, which includes perceptions of children's behavioral problems. Another limitation is that we evaluated multiple aspects of child development using multiple tests, which could result in false-positive significant results. However, we prespecified only 3 primary outcomes and found a significant effect on 1 of these 3, reducing the likelihood that this was due to chance. Also, we observed a differential loss to follow-up between intervention groups, with a higher loss in the non-LNS group. Nonetheless, most maternal baseline characteristics were similar between this sample and the full sample enrolled into the main trial, and the intervention groups in this sample were similar to each other, suggesting a low risk of bias.

To our knowledge, this study is the first to examine the long-term developmental effects of LNS supplementation during both pre- and postnatal periods. Two earlier RCTs examined long-term developmental effects of early supplementary feeding during both pregnancy and early childhood. In Guatemala, 2 villages were assigned to Atole (a high-energy, high-protein supplement) and 2 villages to Fresco (a low-energy, nonprotein supplement) (18). Both supplements were fortified with micronutrients and were targeted at pregnant and lactating women and children up to age 7 y. In Colombia (19), nutritionally at-risk families were randomly assigned to early (pregnancy through 6 mo postpartum) and/or late (6–36 mo postpartum) food supplementation with or without a social stimulation intervention. Effects of supplementation on cognitive function in both studies were observed immediately after the intervention period, with cognitive gains being sustained through school age and beyond. In Guatemala, higher supplement consumption, regardless of assigned group, was associated with higher affect and social involvement, whereas low supplement consumption was associated with passive, despondent, and anxious behavior in children at 6–8 y (40). Social-emotional and behavior problems were not assessed in Colombia. In contrast, whereas we found no immediate positive effects of LNS at age 18 mo on cognitive or social-emotional function, we found differences in social-emotional and behavioral problems at preschool age. At least 2 other studies have found developmental effects of early supplementation on later outcomes, even without finding earlier effects on global developmental assessments (16, 17), consistent with the pattern of results in our cohort. The social-emotional assessment we used at age 18 mo may not have been sensitive enough to detect effects at that early age, when children's emotional and behavioral regulation is immature. Our finding of lower behavior problems in the LNS group at 4–6 y is consistent with the association of higher supplement intake with positive social-emotional and behavioral function at 6–8 y in Guatemala, although those findings were correlational and could have been confounded by unmeasured factors.

It is uncertain why we found no effects on cognition in the present study. In our cohort, the prevalence of baseline maternal anemia (13%) and underweight (2.4%) during pregnancy was low (41). Thus it is plausible that the Ghanaian children were at lower risk of malnutrition during fetal life than the study children in the other 2 trials, and therefore less likely to respond to a nutritional supplement (2). This is consistent with other studies of maternal supplementation during pregnancy and lactation (25, 42, 43) and supplementation of children in early infancy (44, 45) in which treatment effects were found only in subgroups at risk of undernutrition or poor social-economic status. However, the positive effects of the iLiNS-DYAD intervention on birth weight (15) and linear growth at 18 mo (46) show that this population did have potential to benefit from supplementation in some outcomes.

The potential to benefit in social-emotional outcomes may be related to the high prevalence of reported social-emotional difficulties in this sample: about 25% of children had a total difficulties score in the abnormal range based on SDQ standard cutoff scores (≥17) (30, 47), which is high compared with other studies: 3.6% in Denmark (48), 7.1% in Norway (49), and 9.9% in the United Kingdom (47). To our knowledge, our study is the first to document the prevalence of behavioral problems among preschool children in Ghana, and to show that nutritional supplementation decreased the prevalence of parental report of such problems.

Two potential biological explanations for the lower behavioral problem scores in the LNS arm than in the non-LNS arm could be the essential fatty acids provided by LNS and the iron provided to children in the LNS group from 6 to 18 mo. In animal models, DHA deficiency affects brain regions involved in the regulation of emotional status, such as the prefrontal cortex, striatum (50), and dopamine pathways, with accompanying deficits in behavioral and learning tasks (51). Among boys with attention deficit hyperactivity disorder, DHA deficiency has been associated with behavioral and learning problems (52). The LNS provided in our study contained 0.5 g of α-linolenic acid (18:3n−3) (ALA), the omega-3 essential fatty acid precursor to DHA, and 4.6 g of linoleic acid (18:2n−6); the omega-6:omega-3 ratio was thus within the recommended 4–10:1 omega-6:omega-3 fatty acid ratio (53). However, the conversion rate of ALA to DHA is typically low (9% in young women) (54) and we found no effect of maternal LNS supplementation on maternal plasma fatty acid status at 36 weeks of gestation, although breastmilk ALA at 6 mo postpartum was higher in the LNS group than in the non-LNS group among women in this study (55). Thus it is unclear whether the essential fatty acids provided by LNS in early life could account for the difference in child behavior problems at 4–6 y. Iron is needed for neurodevelopmental processes, such as myelination, and for the synthesis of the neurotransmitter dopamine, which is involved in social-emotional regulation (56). Iron deficiency may alter dopamine pathways, which may lead to socioemotional behavioral abnormalities including hyperactivity and inattentiveness (57). Iron deficiency in infancy and early childhood is associated with negative affective behavior, emotion regulation, temperament control, and social and attentional problems in later preschool and school age (58, 59). This suggests that the additional iron provided to the children in the LNS group from 6 to 18 mo is a plausible biological mechanism for the observed effects at 4–6 y.

A third potential explanation is that parents in the LNS group might have had greater expectations for their children's development, given that they were aware of the supplement received, which could have led to biased reports of children's behavioral problems on the SDQ. However, we found no differences between intervention groups in the prosocial skills subscale of the SDQ (also based on the caregiver report), which we would have expected if biased reporting was a factor. In addition, in our cohort, parents’ perceptions of the impacts of the supplements on the index child were equally positive in the LNS and non-LNS groups at follow-up (60), and both groups had high expectations regarding the supplement's impact on the child's future cognitive development and school performance.

In conclusion, the provision of LNS during most of the first 1000-d window decreased behavioral problems reported by caregivers at preschool age, especially for children from low-stimulation households, but did not affect cognition or fine motor function at preschool age in this Ghanaian cohort. Follow-up of this cohort is needed to investigate whether the behavioral effects persist and influence other functional outcomes through school age or young adulthood.

## Supplementary Material

nqy303_Supplemental_File

## Notes

This study was funded by a grant to the University of California, Davis, from the Bill & Melinda Gates Foundation, and the Society for Research in Child Development, 2015 Patrice L Engle Dissertation Grant in Global Early Child Development.

Supplemental Methods and Supplemental Tables 1–5 are available from the “Supplementary data” link in the online posting of the article and from the same link in the online table of contents at https://academic.oup.com/ajcn/.

Abbreviations used: ALA, α-linolenic acid (18:3n−3); HOME, Home Observation for the Measurement of the Environment Inventory; IFA, iron and folic acid; iLiNS, International Lipid-Based Nutrient Supplement; LNS, lipid-based nutrient supplement; MMN, multiple micronutrients; RCT, randomized controlled trial; SDQ, Strengths and Difficulties Questionnaire.
