# Supplementary material for: Prenatal and postnatal lipid-based nutrient supplementation and cognitive, social-emotional, and motor function in preschool-aged children in Ghana: a follow-up of a randomized controlled trial
Source: Am J Clin Nutr. 2019 Feb 5;109(2):322–34. doi: 10.1093/ajcn/nqy303 (PMC6367954; doi:10.1093/ajcn/nqy303)
Supplement: nqy303_Supplemental_File [file nqy303_supplemental_file.docx]

Online Supporting Material: Prenatal and postnatal lipid-based nutrient supplementation and cognitive, social-emotional and motor function in preschool-aged children in Ghana: A follow-up of a randomized controlled trial

**SUPPLEMENTAL METHODS**

*Test selection and adaptation*

We selected a set of motor, cognitive and social-emotional development tests according to the following criteria:

1. High priority criteria: a) ability to distinguish between different children’s abilities, resulting in a good distribution of scores in a sample of children at the target age; b) appropriate for the local setting based on children’s familiarity with test materials and procedures; c) high reliability and validity in the local setting; d) does not require extensive training or subjective judgments by the data collectors; e) relatively brief to administer.

2. Medium priority criteria: a) shown to be sensitive to maternal and child nutritional deficiency in previous studies; b) measuring a wide range of abilities that develop during early childhood, if possible tied to brain systems and mechanisms; c) predicts future outcomes, such as later learning disabilities, cognitive scores, school performance, grade level attained in school or skilled employment, etc.

3. Low priority criteria: a) have established norm values for the target age.

We first developed forms and manuals for each test. We then organized a review and training session with five local field workers. We initially reviewed each test in the Standard English, translated items and instructions into three common local languages (Krobo, Ewe, Twi), and trained on instructions and procedures for assessments. We also discussed appropriate materials and pictures to be used for assessments that are common or normal to the local area.

The tests were adapted to the local setting in Ghana, and the context-specific criteria were evaluated through two rounds of pilot studies conducted in the study area in 2014 and 2015. The pilot study team consisted of an international developmental psychologist, a doctoral student researcher trained in child neurobehavioral assessments (field supervisor), and five trained field workers recruited from the study area who were proficient in the local languages spoken in the area. The process was iterative, involving review and modifications of test materials and pictures to identify cultural appropriateness and acceptable level of difficulty, and personalized interviews with mothers of preschool children in the local area to modify forms to be relevant to children in the local context. For the initial round of pilot testing, test-retest reliability ranged from r = 0.64 - 0.87, with the exception of fine motor and executive function tests and parental reports on social-emotional function (r = 0.34 - 0.58). Internal reliability from Cronbach’s alpha ranged from 0.61 - 0.90 and most tests correlated with age, except for the social-emotional scales. After additional modifications, in the final round of pilot testing, reliability of tests of fine motor and executive function improved (r = 0.61- 0.80) and parent reports on children’s social-emotional function showed higher reliability (r = 0.75 - 0.94).

*HOME Inventory*

The EC version of the HOME (1) is designed for children age 3 to 6 y, containing 55 items clustered into 8 subscales: 1) Learning Materials, 2) Language Stimulation, 3) Physical Environment, 4) Parental Responsivity, 5) Academic Stimulation, 6) Modeling of Social Maturity, 7) Variety in Experience, and 8) Acceptance of child. We made revisions to a few items in the HOME example “parent or other adults in the home buy or read newspapers or magazines regularly” instead of “parent buys and reads a daily newspaper” and also eliminated a few items, for a modified total of 46 items. The total HOME score was calculated as the sum of scores, based on a credit =1 or no credit = 0 scores.

*Maternal depression*

The original 10-item questionnaire was designed as a self-report tool to screen for depressive symptoms postnatal (2) . We used a cut off score of 12 or greater, out of a total score of 30, to indicate mothers showing depressive symptoms in our sample.

*Maternal Agency*

The General self-efficacy scale was designed to help predict coping mechanisms adapted towards stressful life events (3). Mothers or caregivers were asked ten questions about how they are successfully coping in the face of different challenges and situations in life, setting goals, investing efforts into the future, and recovering from drawbacks in the past. Scores were the sum of points on all ten items with responses ranging from 1 to 4 from little or no success in coping to high success.

*Behavior rating scale*

Items selected from the Leiter-R subscales include: attention (e.g., pays attention during instructions), activity level (e.g., remains in seat), sociability (e.g., alert and interactive), and energy and feelings (e.g., shows pleasure in accomplishment). In addition, a few items were included from the DB-DOS coding system to capture defiance and noncompliance, negative and positive affect and verbal or physical aggression. One item was also included to capture children’s anxiety during the assessment (“shows frequent/intense feelings of worry”). Items were coded using a Likert scale ranging from 0 to 3 and some items were reverse-coded to minimize automatic responding.

*Training of data collectors*

On average, we recorded high inter-rater agreement over the year for: head-toe: 95%; PEDS: 91%; paired associate: 98%; comprehension of instructions: 92%; body part naming and identification: 97%; pegboard: 90%; behavior rating: 90%; block design: 92% and delay of gratification: 100%.

**SUPPLEMENTAL RESULTS**

*Effect of the intervention: Post-hoc exploratory analysis of SDQ social-emotional difficulties subscale*

In a post-hoc exploratory analysis, we assessed the effect on the intervention on each of the 4 subscales that make up the SDQ total difficulties score: emotional symptoms z-score, conduct problems z-score, hyperactivity z-score, and peer relationships problems z-score.

In unadjusted analysis (model 1), the LNS group showed a trend for a lower mean peer problems score than the non-LNS group (Mean ± SE: LNS = -0.08 ± 0.05; non-LNS = 0.04 ± 0.04 p = 0.067). With adjustment for baseline and follow-up covariates (model 3), this difference was also marginally significant (p = 0.056).

In unadjusted analysis we found no significant differences between groups on the conduct problems score (Mean ± SE: LNS = -0.06 ± 0.05; non-LNS =

0.03 ± 0.04 p = 0.154). However, with adjustment for baseline and follow-up covariates the LNS group showed a trend for a lower mean conduct problems score than the non-LNS group (p = 0.083).

We found no significant difference between the two groups in hyperactivity and emotional symptoms (ps > 0.1), however a pattern of effect modification found with the overall SDQ total difficulties score was replicated for the emotional problems score; the effects of LNS was greater in children from low nurturing and stimulation homes (p-interaction = 0.045).

**REFERENCES FOR SUPPLEMENTAL MATERIAL**

1. Caldwell B, Bradley R. Home Observation for Measurement of the Environment: Administration Manual. . Tempe, AZ: Family & Human Dynamics Research Institute, Arizona State University Google Scholar 2003.

2. Cox JL, Chapman G, Murray D, Jones P. Validation of the Edinburgh Postnatal Depression Scale (EPDS) in non-postnatal women. Journal of affective disorders 1996;39(3):185-9.

3. Schwarzer R, Jerusalem, M. Generalized Self-Efficacy scale. Edtion ed. In: J. Weinman SW, M. Johnston ed. Measures in health psychology: A user’s portfolio Causal and control beliefs. Windsor, UK: NFER-NELSON, 1995:35-7.

**SUPPLEMENTAL TABLES**

**Supplemental Table S1: Background characteristics of all children enrolled in original trial vs children tested at follow-up**

|  | **Tested at follow-up** | **Lost to follow-up** |  |
| --- | --- | --- | --- |
| Variable | Mean ± SD [n] or % [n/total] | Mean ± SD [n] or % [n/total] | p-value |
| Baseline Maternal age (y) | 26.8 ± 5.4 [966] | 26.5 ± 5.7 [354] | 0.329 |
| Pre-Pregnancy BMI^1^ (Kg/m^2^) | 24.6 ± 4.5 [949] | 24.4 ± 4.1 [342] | 0.871 |
| Gestational age at enrolment (wk) | 16.1 ± 3.3 [966] | 16.1 ± 3.3 [345] | 0.879 |
| Baseline Maternal education (y) | 7.6 ± 3.5 [966] | 7.7 ± 3.9 [354] | 0.528 |
| Baseline Maternal hemoglobin concentration (g/L) | 111.3 ± 12.0 [965] | 111.6 ± 12.3 [354] | 0.682 |
| Baseline Household asset score^2^ | 0.010 ± 1.0 [953] | -0.010 ± 1.1 [329] | 0.758 |
| Nulliparous (%) | 32.1 [310/966] | 38.4 [136/354] | 0.032 |
| Gestational age at delivery (wk) | 39.3 ± 0.0 [961] | 39.1 ± 0.1 [282] | 0.234 |
| Male (%) | 47.8 [461/965] | 53.2 [150/282] | 0.110 |
| Mean maternal adherence pregnancy - 6 mo postpartum (% of supplements consumed) | 73.1 [697/954] | 50.2 [140/279] | <0.0001 |

LNS = Lipid-based Nutrient Supplement. Non-LNS = Iron & folic acid + multiple micronutrient capsules (control group). Results are based on ANOVA (SAS PROC GLIMMIX) or Chi-square (SAS PROC FREQ)^. 1^Estimated pre-pregnancy BMI was calculated from estimated pre-pregnancy weight (based on polynomial regression with gestational age, gestational age squared, and gestational age cubed as predictors) and height at enrollment. ^2^Proxy indicator for household socioeconomic status constructed for each household based on ownership of a set of assets (radio, television etc.), lighting source, drinking water supply, sanitation facilities, and flooring materials. Household ownership of this set of assets is combined into an index (with a mean of zero and standard deviation of one) using principal components analysis. Higher value represents higher socioeconomic status.

| **Supplemental Table S2: Selected characteristics of women and children by intervention group at baseline & follow-up** | | | | |
| --- | --- | --- | --- | --- |
|  | **IFA**  n=299 | **MMN**  n=327 | **LNS**  n=340 |  |
| Variable | Mean ± SD [n] or % [n/total] | Mean ± SD [n]  or % [n/total] | Mean ± SD [n]  or % [n/total] | p-value |
| Baseline maternal age (y) | 26.6 ± 5.2 [299] | 26.9 ± 5.6 [327] | 26.9 ± 5.5 [340] | 0.755 |
| Pre-pregnancy BMI^1^ (kg/m2) | 24.2 ± 4.3 [294] | 24.6 ± 4.7 [319] | 24.8 ± 4.4 [336] | 0.123 |
| Gestational age at enrolment (wk) | 16.1 ± 3.3 [299] | 16.1 ± 3.2 [327] | 16.1 ± 3.3 [340] | 0.995 |
| Baseline maternal education (y) | 7.7 ± 3.3 [299] | 7.4 ± 3.6 [327] | 7.6 ± 3.7 [340] | 0.556 |
| Baseline maternal hemoglobin conc. (g/L) | 112.2 ± 13.2 [299] | 110.6 ± 11.6 [326] | 111.2 ± 11.3 [340] | 0.261 |
| Baseline household asset score^2^ | 0.04 ± 0.96 [298] | 0.08 ± 0.95 [322] | -0.09 ± 0.97 [334] | 0.062 |
| Nulliparous (%) | 35.5 [106/299] | 28.8 [94/327] | 32.4 [110/340] | 0.199 |
| Gestational age at delivery (wk) | 39.2 ± 0.1 [298] | 39.4 ± 0.1[325] | 39.4 ± 0.10 [338] | 0.634 |
| Child male (%) | 49.2 [147/299] | 45.9 [150/327] | 48.2 [164/340] | 0.717 |
| Child age at follow-up (y) | 4.9 ± 0.0 [299] | 4.9 ± 0.0 [327] | 5.0 ± 0.0 [340] | 0.076 |
| Mean maternal adherence pregnancy -6 mo postpartum (% of supplements consumed) | 77.0 [227/295] | 75.6 [245/324] | 67.2 [225/335] | 0.010 |
| Home stimulation score at follow-up | 27.7 ± 4.8 [285] | 28.1 ± 4.9 [314] | 27.9 ± 4.5 [329] | 0.653 |
| LNS = Lipid-based Nutrient Supplement. IFA = Iron & folic acid capsules. MMN = multiple micronutrient capsules. Results are based on ANOVA (SAS PROC GLIMMIX) or Chi-square (SAS PROC FREQ). ^1^Estimated pre-pregnancy BMI was calculated from estimated pre-pregnancy weight (based on polynomial regression with gestational age, gestational age squared, and gestational age cubed as predictors) and height at enrollment. ^2^Proxy indicator for household socioeconomic status constructed for each household based on ownership of a set of assets (radio, television etc.), lighting source, drinking water supply, sanitation facilities, and flooring materials. Household ownership of this set of assets is combined into an index (with a mean of zero and standard deviation of one) using principal components analysis. Higher value represents higher socioeconomic status. | | | | |

| **Supplemental Table S3: Age-adjusted primary outcomes at 4-6 y by intervention group, and comparison of treatment effects between groups** | | | | | | | | | | | |
| --- | --- | --- | --- | --- | --- | --- | --- | --- | --- | --- | --- |
|  | | | | | | **Comparison of MMN and IFA** | | **Comparison of LNS and IFA** | | **Comparison of LNS and MMN** | |
| **Domain (z-scores)** | n | **IFA**  **Mean**  **(95% CI) or OR [n/total]** | **MMN**  **Mean**  **(95% CI) or OR [n/total]** | **LNS**  **Mean**  **(95% CI) or OR [n/total]** | **P-value** | **Difference in mean or OR (95% CI)** | **P-value** | **Difference in mean or OR (95% CI)** | **P-value** | **Difference in mean or OR (95% CI)** | **P-value** |
| Cognitive z-score | 951 | -0.03 (-0.13, 0.07) | 0.01  (-0.09, 0.11) | 0.03  (-0.06, 0.13) | 0.696 | 0.04  (-0.13, 0.21) | 0.339 | 0.06  (-0.11, 0.23) | 0.676 | -0.02  (-0.19, 0.14) | 0.948 |
| Motor z-score | 963 | 0.04 (-0.06, 0.15) | -0.03  (-0.13, 0.07) | 0.0  (-0.10, 0.09) | 0.603 | -0.07  (-0.24, 0.10) | 0.576 | -0.04  (-0.21, 0.13) | 0.821 | -0.03  (-0.19, 0.14) | 0.907 |
| Social-emotional difficulties z-score | 958 | 0.04 (-0.07, 0.15) | 0.04  (-0.07, 0.15) | -0.08  (-0.18, 0.03) | 0.231 | 0.00  (-0.19, 0.19) | 0.100 | -0.12  (-0.30, 0.07) | 0.309 | 0.1  (-0.07, 0.30) | 0.299 |
| LNS = Lipid-based Nutrient Supplement. IFA = Iron & folic acid capsules. MMN = multiple micronutrient capsules. Results are based on ANCOVA (SAS PROC GLIMMIX). | | | | | | | | | | | |

| **Supplemental Table S4: Non-continuous outcome: Predicted probability of delaying gratification at 4-6 y** | | | | | | | | |
| --- | --- | --- | --- | --- | --- | --- | --- | --- |
|  |  | **IFA** | **MMN** | **LNS** | **MMN**  **vs**  **IFA** | **LNS**  **vs**  **IFA** | **p-value adjusted for child age at follow-up** | **p-value adjusted for baseline and other covariates** |
| **# of Treats** | **N^1^** | **Predicted probability^2^ (95% CI)** | | | **OR (95% CI)^3^** | |  |  |
| 4 | 242 | 0.274  (0.230, 0.317) | 0.305  (0.228, 0.381) | 0.337  (0.221, 0.454) | 1.294  (0.959, 1.746) | 1.067  (0.790, 1.440) | 0.205 | 0.285^4^ |
| 3 | 63 | 0.070  (0.065, 0.075) | 0.074  (0.066, 0.081) | 0.077  (0.067, 0.087) |  |  |  |  |
| 2 | 153 | 0.166  (0.161, 0.170) | 0.167  (0.163, 0.171) | 0.169  (0.178, 0.161) |  |  |  |  |
| 1 | 503 | 0.491  (0.439, 0.543) | 0.455  (0.367, 0.543) | 0.416  (0.299, 0.534) |  |  |  |  |
| LNS = Lipid-based Nutrient Supplement. IFA = Iron & folic acid capsules. MMN = multiple micronutrient capsules. ^1^Sample size based on model 1 adjusted for child age at follow-up. Results are based on logistic regression (SAS PROC GLIMMIX). ^2^ Probabilities modeled are cumulated over the lower ordered values. Probabilities of membership to the categories of treats estimated with intercept for lower ordered values, intervention group and median child age. ^3^Odds ratio: Odds of a one category higher response in LNS/MMN vs the odds of a one category higher response in IFA. ^4^Adjusted for child age at follow-up, maternal agency and maternal depression. | | | | | | | | |

| **Supplemental Table S5: Secondary outcomes: Selected developmental outcomes at 4-6 y by intervention group^1^, and adjusted model** | | | | | | | | | |  |
| --- | --- | --- | --- | --- | --- | --- | --- | --- | --- | --- |
|  | | | | **Adjusted for child age at follow-up^3^** | | **Adjusted for baseline covariates^4^** | | **Adjusted for baseline and other covariates^5^** | | **Covariates** |
| **Domain**  **(z-scores)** | **N^2^** | **LNS**  **Mean (95% CI) or OR [n/total]** | **Non-LNS**  **Mean (95% CI) or OR [n/total]** | **Difference in mean or OR (95% CI)** | **P-value** | **Difference in mean or OR (95% CI)** | **P-value** | **Difference in mean or OR (95% CI)** | **P-value** |  |
| Language z-score | 963 | 0.00  (-0.08, 0.09) | 0.00  (-0.06, 0.06) | 0.00  (-0.10, 0.11) | 0.936 | 0.02  (-0.08, 0.12) | 0.702 | 0.01  (-0.09, 0.11) | 0.796 | A,B,F,G,H,M,N |
| Visuospatial z-score | 963 | -0.02  (-0.08, 0.04) | 0.00  (-0.04, 0.04) | -0.02  (-0.09, 0.06) | 0.675 | -0.02  (-0.09, 0.05) | 0.612 | -0.01  (-0.09, 0.08) | 0.853 | B,C,G,I,K,N |
| Pre-academic z-score | 958 | -0.01  (-0.12, 0.10) | 0.00  (-0.08, 0.08) | -0.01  (-0.15, 0.12) | 0.852 | 0.00  (-0.12, 0.13) | 0.965 | -0.01  (-0.15, 0.14) | 0.927 | A,B,C,D,E,G,H,I,J,K,M,N,O |
| Declarative memory z-score | 958 | 0.04  (-0.06, 0.14) | -0.03  (-0.10, 0.04) | 0.07  (-0.06, 0.19) | 0.285 | 0.08  (-0.04, 0.20) | 0.209 | 0.04  (-0.09, 0.17) | 0.568 | A,B,E,F,H,I,N |
| Behavior rating z-score | 962 | 0.00  (-0.11, 0.10) | 0.0  (-0.08, 0.08) | -0.01  (-0.14, 0.12) | 0.910 | -0.01  (-0.14, 0.12) | 0.922 | -0.01  (-0.15, 0.13) | 0.884 | B,G,H,I,M,N |
| Cognitive lowest decile | 951 | 10.2 [34/335] | 10.1 [62/616] | 0.99  (0.64, 1.55) | 0.972 | 0.95  (0.60, 1.51) | 0.834 | 1.05  (0.59, 1.86) | 0.880 | B,E,G,J,L,N |
| Cognitive lowest quartile | 951 | 24.5 [82/335] | 25.3 [156/616] | 0.95  (0.70, 1.30) | 0.761 | 0.93  (0.67, 1.28) | 0.637 | 1.01  (0.71, 1.44) | 0.952 | A,B,C,E,H,I,M,N |
| Social-emotional problems highest decile | 958 | 8.9 [30/336] | 10.9 [68/622] | 0.80  (0.51, 1.26) | 0.332 | 0.74  (0.47, 1.18) | 0.208 | 0.64  (0.39, 1.06) | 0.085 | H,M,N,O |
| Social-emotional problems highest quartile | 958 | 20.8 [70/336] | 27.8 [173/622] | 0.68  (0.49, 0.93) | 0.016 | 0.65  (0.47, 0.90) | 0.010 | 0.61  (0.43, 0.88) | 0.008 | A,B,C,E,H,M,N,O |
| Motor lowest decile | 963 | 11.2 [38/338] | 9.4 [59/625] | 1.23  (0.80, 1.89) | 0.355 | 1.24  (0.80, 1.89) | 0.333 | 1.23  (0.75, 2.02) | 0.404 | G,I,N,O |
| Motor lowest quartile | 963 | 25.4 [86/338] | 24.8 [155/625] | 1.02  (0.75, 1.39) | 0.879 | 1.02  (0.75, 1.39) | 0.885 | 1.03  (0.73, 1.46) | 0.853 | B,G,I,N,O |
| \| LNS = Lipid-based Nutrient Supplement. IFA = Iron & folic acid capsules. MMN = multiple micronutrient capsules. Results are based on ANCOVA (SAS PROC GLIMMIX). ^1^We first tested the null hypothesis of no difference between the 3 treatment groups, and combined the IFA/MMN groups because there were no significant differences between those 2 groups.  ^2^Sample size based on model 1 adjusted for child age at follow-up. ^3^All models were adjusted for child age at follow-up. ^4^Baseline covariates=A-F. ^5^Other covariates collected after baseline= G-O. A= maternal age; B= maternal education; C= maternal hemoglobin; D= maternal pre-pregnancy BMI; E= household asset score; F= primiparity; G= child sex; H= data collector; I= exposure to multiple languages by 18 mo; J= type of preschool; K= teacher’s education; L= average time in instructions at preschool; M= maternal depression; N= home stimulation score; O= maternal agency. \| \| --- \| | | | | | | | | | | |
